# Clinical System for Mood Disorder Care in Córdoba, Colombia: Participatory Design and Scenario-Based Usability Evaluation Study

**DOI:** 10.2196/58909

**Published:** 2025-10-06

**Authors:** Ever Augusto Torres-Silva, Juan José Gaviria-Jiménez, Eider Pereira-Montiel, David Andrés Montoya-Arenas, José Fernando Flórez-Arango

**Affiliations:** 1 Bioengineering and Microelectronics Research Group School of Medicine Universidad Pontificia Bolivariana Medellín Colombia; 2 Informed School of Medicine Universidad de Antioquia Medellín Colombia; 3 GI2B Research Group Department of Exact and Applied Sciences Instituto Tecnológico Metropolitano Medellín Colombia; 4 Psychology Research Group School of Social Sciences Universidad Pontificia Bolivariana Medellín Colombia; 5 Department of Population Health Sciences Division of Health Informatics and Artificial Intelligence Weill Cornell Medical College New York, NY United States

**Keywords:** user-centered design, user-computer interface, workload, mental health, mental health services, mood disorders, telehealth

## Abstract

**Background:**

Mood disorders are among the leading causes of disability worldwide and present a growing public health concern. In Córdoba, Colombia, suicide rates have risen significantly in recent years, exposing structural gaps in mental health care delivery. Digital health solutions and telehealth interventions can expand access to early detection, referral, and monitoring of patients in underserved regions. However, their effectiveness depends on rigorous and diverse evaluations to ensure adoption and sustainability.

**Objective:**

This study evaluated the usability of a clinical telehealth system for mood disorder care developed through participatory design, with emphasis on user-centered functionality and workload analysis.

**Methods:**

The system was designed through 2 iterative development cycles, followed by a scenario-based usability evaluation. A functional Domain Ontology was constructed to prioritize 8 core functionalities, including telecounseling, a georeferenced institutional directory, hotline services, patient self-report tools, educational content, forums, and a population dashboard. Thirty participants representing patients, caregivers, clinical staff, and administrative personnel were recruited through convenience sampling. Usability was assessed through cognitive walk-throughs, the NASA (National Aeronautics and Space Administration) Task Load Index, and the Post-Study System Usability Questionnaire.

**Results:**

A total of 34 usability sessions and 223 task-level workload assessments were conducted across 2 evaluation cycles. The system demonstrated high usability, with overall Post-Study System Usability Questionnaire scores of 2.2 in cycle 1 and 2.3 in cycle 2. Interfaces prioritized for patients and clinical staff achieved better evaluations (average 1.9-2.0) than administrative interfaces (average 3.0). Workload analysis indicated improvements between cycles, particularly for patient-centered tasks, with mental workload as the most significant source of cognitive demand. Twenty-three critical issues (9 system errors and 14 design flaws) were identified and corrected between cycles, leading to measurable usability gains.

**Conclusions:**

The participatory and scenario-based approach facilitated early identification of usability challenges and supported iterative refinement of the system. Results suggest that the system is usable, acceptable, and effective in reducing workload for key user groups, particularly patients and clinicians. The findings reinforce the value of participatory methodologies in digital mental health and highlight the need to prioritize patient-facing interfaces. Future research should extend evaluations to mobile platforms and larger populations to support scalability and integration into regional mental health services.

## Introduction

Recent studies indicate that mental health problems have reached epidemic proportions, with disorders such as depression, anxiety, bipolar disorder, trauma-related disorders, and stressors contributing significantly to the global burden of disease [1,2]. Epidemiological research reports that these conditions affect a large proportion of the world's population, often resulting in chronic disability and reduced quality of life. In parallel, there is growing evidence that high levels of alcohol consumption play a critical role in both precipitating mental health disorders and exacerbating symptoms in those already affected [3]. Despite the widespread prevalence and considerable individual and societal impact of these disorders, a treatment gap persists. Less than half of those with mental illness receive adequate care, a deficit attributed to stigma, limited financial resources, and deficiencies in existing health care infrastructures [4,5]. In the Colombian department of Córdoba, Simancas Fernández et al [6] conducted a review of 33 sources describing the mental health situation of the local population. Their findings evidenced a 33% increase in suicide cases between 2020 and 2022, identifying risk factors such as gender, depressive and anxiety symptoms, substance abuse, history of suicide, family dysfunction, forced displacement, and experiences of bullying [6]. Faced with the challenges posed by mental disorders, public policy makers and health professionals are increasingly advocating for the implementation of integrated care models. In this context, telehealth and the use of digital technologies emerge as key strategies to enhance remote detection and monitoring of patients by specialized health care personnel, aiming to improve access for populations in underserved areas, optimize patient follow-up, and reduce unnecessary travel, among other benefits [7].

An adequate participatory design of technological tools and high usability contributes to the improvement of the use of such strategies and enhances adherence, user satisfaction, and development and maintenance costs [8]. Participatory design consists of including the system’s end users during the process. This participation is carried out in all design phases [9]. Usability evaluation of a product or system refers to the process of identifying how users complete activities and can meet the intended (usefulness) objectives while doing so easily and with a low level of errors [10]. There are multiple evaluation methods, including heuristic evaluation, scenario evaluation, and focus groups. These processes evaluate and apply rules to improve the usability of systems and tools both in the health software engineering and user-centered design area and in the care and clinical scenarios, for example, the design of electronic medical record systems, the design of mobile apps, and the adjustments and design of biomedical devices [11]. Recent studies support these approaches: in the development of the Kuamsha app for behavioral activation therapy, the authors implemented an iterative, user-centered agile design methodology involving adolescents in all design phases through focus groups, in-depth interviews, participatory workshops, and multicycle usability testing sessions to refine the interface, narrative elements, and functionality based on direct user feedback [12]. Similarly, the participatory design of a conversational artificial intelligence agent for mental health care incorporated focus groups with both users and psychotherapists during the early prototyping phases; usability and engagement were evaluated qualitatively through therapist feedback and patient-reported experiences after app use, which guided iterative improvements to ensure the system’s integration into psychotherapy workflows [13].

Furthermore, recent work highlights that addressing quality, usability, and user engagement from the outset is essential for the long-term success of eHealth interventions. A cocreation-based framework, in which users, health care professionals, and developers collaborate as equal partners, has been proposed as an effective strategy to ensure usability, personalization, and sustained behavior change, particularly when artificial intelligence tools dynamically adapt content to user needs [14]. Building upon this evidence, this research aimed to evaluate the usability level of a system built through participatory design for detecting mood disorders and supporting telehealth care in mental health.

## Methods

### Study Design

**Figure 1:**
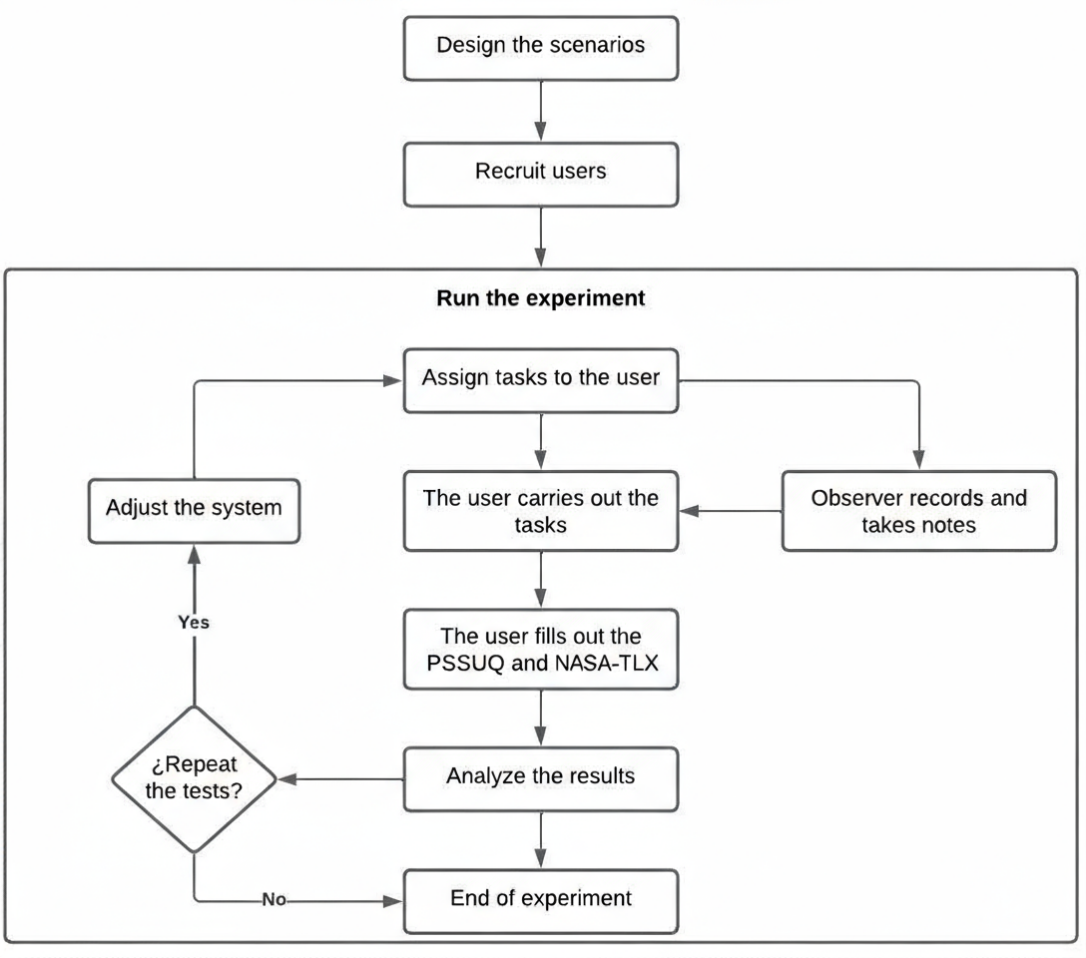
Scenario-based usability evaluation flow applied in a participatory design study of a clinical information system for the care of people with mood disorders in Córdoba, Colombia (2022-2023). The diagram illustrates the sequence of steps, including scenario design, user recruitment, task assignment, observation, completion of PSSUQ and NASA-TLX questionnaires, system adjustments, and repeated testing across 2 development cycles. NASA-TLX: National Aeronautics and Space Administration Task Load Index; PSSUQ: Post-Study System Usability Questionnaire.

The study follows a participatory design approach that required 2 iterations of development followed by a scenario-based evaluation. This methodology allows the identification of bottlenecks, additional system failures, ease of learning the tool, and the comprehensibility of the language, among others [15]. In the evaluation methodology by scenarios, the main functionalities and tasks included in the mental health care model of the Department of Córdoba, Colombia, were included. The Department of Córdoba is located in the northwestern region of Colombia, covering an area of approximately 25,020 km² (9660 square miles) and home to around 2 million people as of 2025 [16]. This model includes a system designed to provide a service for the timely detection of mood disorders, contact with mental health care personnel, and support for referring cases to the network of health care providers. At the time of the evaluation, the system had been built through participatory design and had not been released to end users. The study had three phases: (1) test scenario design and creation phase, (2) test user recruitment phase, and (3) test implementation phase (Figure 1).

### Scenario Preparation and Creation Phase

Functionalities and scenarios to be evaluated were selected based on the Knowledge Domain Ontology, following the GOMS (Goals, Objectives, Methods, Selection rules) methodology [11]. Two activities were carried out to construct the functional Domain Ontology (DO): (1) cocreation exercise with the project’s coresearchers (16 psychologists, biomedical engineers, and communicators), in which the contributions of each of them were compiled on a collaborative platform; and (2) visits to governmental actors to identify the specific needs of the territory. This information was synthesized in Microsoft Excel. As a result, 8 basic functionalities of the system, containing 60 specific tasks, were found: (1) Telecounseling: the possibility of interacting with expert mental health personnel for patients and caregivers. (2) Directory of institutions: identification of georeferenced institutions that are part of the program. (3) Hotline: access through multiple communication channels to emergency mental health care. (4) Forum: the possibility of exchanging experiences among users of the mental health program. (5) Questions: compilation and display of frequently asked questions for quick reference. (6) Self-reported questionnaires: the possibility of completing scales, surveys, and screening and diagnostic tools reported to expert mental health personnel. (7) Information capsules: educational content about mental health and its care. (8) Population dashboard: display of active patients in the mental health program.

DO allows for prioritization of functionalities to be implemented through the frequency analysis of the potential users’ participation in the tasks identified in the functional analysis described in the section “Scenario Preparation and Creation Phase.” The DO identified main user groups: (1) Patients: individuals interested in receiving mental health services. Some of them formally join the program, which requires full identification of the individuals. These users have control over access to their own information. (2) Caregivers: individuals interested in getting mental health services for their care recipients. They may or may not be identified, and some formally join the program, which requires full identification of the individuals. Access to patient information is limited. (3) Clinical staff: personnel trained at different levels of education to support the diagnosis, treatment, rehabilitation, and monitoring of individuals with mental health conditions. It ranges from community health workers to highly specialized personnel. (4) Administrative personnel: staff responsible for coordinating the service provision and performing administrative activities such as monitoring, billing, scheduling services, and managing demand, among others. (5) Departmental Health Secretariat: the governmental body responsible for directing, coordinating, and administering public health actions across the department, including the oversight and integration of a network of public and private health care service providers.

For the purposes of the study, the patient and caregiver groups were merged into a patient group since caregivers’ tasks constituted a subset of patient tasks. Although the Departmental Health Secretariat was initially identified as a key stakeholder, it was not possible to include their participation in the study, as no officials were assigned to collaborate with the research activities.

### User Recruitment Phase

The sample selection followed a convenience sampling. Potential users were recruited in 2 phases (September and October 2023) by the teaching team of the Faculties of Psychology of Universidad Pontificia Bolivariana at their sites in Medellín, Montería, and Palmira. The 3 types of roles (patient, clinical staff, and administrative personnel) with the greatest participation in the use of the mental telehealth system were included.

The inclusion criteria for the different types of participants were as follows: (1) *Patients and relatives* of patients older than 18 years (legal age in Colombia). (2) *Mental health care personnel* who were psychology or social work students in semesters of clinical internship (ninth semester of the degree or greater) or professionals in psychology or social work. (3) *Administrative or logistical* personnel with responsibilities in managing the population health program.

Potential users who did not have basic computer skills or who required support from others were excluded. After the initial call by the teaching team, the research team contacted the test participants to explain the evaluation procedure, and the synchronous testing spaces were coordinated through the Microsoft Teams platform. Participation was voluntary with verbal consent. The only data collected from individuals were those associated with the features of the type of user (age, gender, level of education, and occupation), excluding data that would allow their identification. The sessions were recorded on the Teams platform in case additional analysis was needed, and access was restricted to the research group.

### Implementation Phase and Usability Evaluation by Scenarios

The scenario-based evaluation methodology was used, accompanied by cognitive walk-throughs observations, in which the user expressed his or her actions, thoughts, and interpretations of the tool aloud [17,18]. The usability tests were conducted individually, and the permissions and access to the respective screens and interfaces were verified at the beginning of each test. Moreover, a document with instructions and proposed tasks to be performed was shared with the users.

To understand different sources of tasks performance in the solution and provide detailed feedback on the need to improve the interface, we conducted task analysis, followed by usability analysis. After each scenario, each user filled out a Google Forms survey containing a questionary for task load measurement (the NASA [National Aeronautics and Space Administration] Task Load Index [NASA-TLX]) [19]. At the end of all the assigned scenarios, users filled out the Post-Study System Usability Questionnaire (PSSUQ) [20].

#### NASA Task Load Index

It is a self-administered questionnaire that seeks to measure the sources of workload and generates a load index that allows observing how a system affects the load of an individual when completing tasks during its use. This questionnaire has 2 parts: the first establishes the magnitude of each factor on a scale from 1 (low) to 10 (high), and the second makes a paired comparison to determine which factor is more important than another.

The factors contained in the scale are (1) Mental demand (M refers to the level of mental demand required by the task), (2) Physical demand (P refers to the level of physical demand), (3) Temporal demand (T refers to the pace imposed by the task), (4) Performance (P refers to the perceived performance in completing the task), (5) Effort (E refers to the level of effort and difficulty of the task), and (6) Frustration (F refers to how insecure, irritated, stressed, or annoyed one felt with the task) [19].

The NASA-TLX index is calculated as follows:



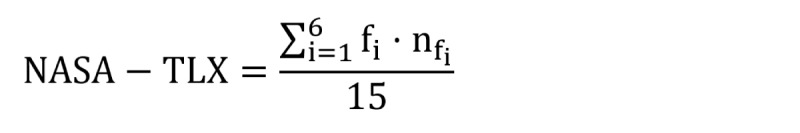



where is a constant indicating the number of factors, is the weighted loading source corresponding to a value assigned to each factor , corresponds to the number of times a factor is selected, and 15 is the number of possible comparisons between factors.

#### Poststudy System Usability Questionnaire

It is a self-managed questionnaire that seeks to measure the level of usability and satisfaction of users after interacting with interfaces or applications. Version 3 is composed of 16 items, which are scored on a Likert scale with 7 options, with 1 being Strongly Agree and 7 Strongly Disagree.

The global score of the scale is calculated with the average of all the scores obtained from the 16 statements or items. It also allows the calculation of three subscales, as follows: (1) System Usefulness (SYSUSE), which is the average of items 1- 6; (2) Information Quality (INFOQUAL), which corresponds to the average of items 7-12, and (3) Interface Quality (INTERQUAL), which is the average of items 13-15. These subscales provide more information and details about aspects of the software or system [20]. For the interpretation process, low scores refer to better levels of usability. According to Sauro and Lewis [21], the following score limits were considered adequate levels: SYSUSE 2.80, INFOQUAL 3.02, INTERQUAL 2.49, and overall average 2.82.

A researcher (EPM) was present during all the tests through teleconference spaces in order to provide support and assistance without interfering in the evaluation activities. During the tests, the user evaluator reported the findings, and the accompanying researcher (EPM) recorded the application errors identified. Findings were consolidated in a project management platform (Azure DevOps), which served as input for the technical and development team of the evaluated mental health system. All the application errors identified during the testing scenarios were corrected for the second evaluation cycle.

### Ethical Considerations

This study was reviewed and approved by the health research ethics committee of Universidad Pontificia Bolivariana, Medellín, Colombia (Minute 09 of 2022). All participants were informed of the objectives and procedures of the research prior to their involvement, and verbal informed consent was obtained before initiating the usability testing sessions. Participation was voluntary. To ensure privacy and confidentiality, no personally identifiable information was collected. Only general demographic characteristics (age, gender, level of education, and occupation) were recorded. Usability sessions were conducted via Microsoft Teams and recorded exclusively for research purposes, with access restricted to the research team. All data were anonymized prior to analysis. Participants did not receive any financial or in-kind compensation for their involvement. No identifiable images of participants are included in this manuscript or in supplementary materials.

## Results

### Scenario Preparation and Creation Phase

The summary of the scenarios created is shown in Table 1. Patients, clinical staff, and system administrators were identified as potential users. As shown, patients are the ones with the most types of interactions anticipated in the system.

**Table 1:** Summary of test scenarios by user role during the scenario-based usability evaluation of a participatory-designed clinical information system for mood disorder care in Córdoba, Colombia (2022-2023)^a^.

Scenario			Scenario name		Statement
Patients					
	1	Login		You are a user who wants to register in the program, and you create an account in the system.	
	2	View directory		You are a user of the system and want to see the list and location of the nearest mental health care providers.	
	3	Call to the hotline		You are not feeling well today. You are having suicidal thoughts and want to contact the mental health hotline by telephone.	
	4	Emergency WhatsApp		You are not feeling well today. You are having suicidal thoughts and want to contact the mental health hotline via WhatsApp (no need to type a message).	
	5	Make an appointment		You want to make an appointment (psychology) of telecounseling by videoconference for today with the professional Eider Pereira.	
	6	Start the appointment		You have an appointment scheduled for today and will attend by videoconference.	
	7	Data entry (scale)		You are concerned about your mental health; you access the system and fill out the DASS-21 questionnaire.	
	8	See the information capsules		You are a patient and want to know about depression in general. Your task is to find the information in the system.	
	9	Start forum post		You are a user and want to make the following post in the forum: Title: How I Feel Experience Description: Hello, my name is XXX. After seeing this space, I feel encouraged to share that many times I have felt down and stressed due to the large number of school assignments and exams. Studying for hours takes up a lot of time and prevents me from doing other activities I enjoy. When exams are approaching, I tend to eat more, sleep less, and even bite my nails a lot. I sometimes feel like running away and leaving everything behind. My parents constantly remind me that I have to be the best and that sacrifices are necessary. They are very concerned about my academic performance. I have few friends at school, and I would like to have more, but I am afraid they might not like me or want me to be part of their group. I don't know if others experience the same, but I often feel bored, unmotivated, and overwhelmed by the responsibilities I am expected to fulfill. I know that stress affects me a lot, and I want to find a way to feel better. Has anyone gone through something similar? What do you recommend I do when I feel this way? Thank you for your attention. Category: Experiences	
	10	Forum comments		You are a patient or user and would like to comment on the forum post called “Mis formas de afrontar la depresión” (My ways of coping with depression, in English). Comment: Thanks for sharing it with the community	
	11	Questions and answers		You are a user of the system, you have the following question about mental health and you ask it in the question section: What is depression?	
Clinical staff					
	1	View agenda		You are a therapist and want to view the list of patients scheduled for today.	
	2	Register service		You are tasked with moderating the mental health forum by approving or rejecting pending posts.	
	3	Moderate the forum		You are tasked with moderating the mental health forum by approving or rejecting pending posts.	
	4	Questions and answers		You are going to answer the following user-submitted question pending approval: “What is depression?” Answer: Depression is an illness that affects your mental state, thereby altering your daily life. It is often characterized by persistent feelings of sadness and a loss of interest in activities such as eating, working, or studying, as well as disruptions to sleep patterns, including insufficient or nonrestorative sleep. These symptoms must persist for at least 4 weeks to be considered depression. Management of depression requires a multidisciplinary approach, which, depending on the patient’s condition, may involve pharmacological treatment and/or psychotherapy. However, only a psychiatrist can establish the appropriate management plan, so I recommend scheduling a medical appointment with this professional. Additionally, I would like to understand why you are experiencing difficulties communicating with your mother. It is crucial to seek emergency care if you are unable to sleep at all or have thoughts that your life has no meaning. Please seek help from a psychiatric professional as soon as possible. (Excerpt from 1DOC3). Category: Depression	
Administrative					
	1	Create the information capsule		You are a provider leader in the system and want to update and add the following information in the capsule module: Image: Any appropriate image can be placed here. Title: How Do I Know If I Am Stressed? Tags: Stress symptoms, Recommendations Description: Stress symptoms can affect your body, thoughts, feelings, and behavior. The most common symptoms associated with stress include headaches, muscle tension or pain, chest pain, fatigue, changes in sex drive, upset stomach, and sleep disturbances. If you have experienced more than one of these symptoms in the past week, it is very likely that you are experiencing signs of stress. Therefore, we encourage you to continue learning about stress management and to seek professional help if necessary. Category: Stress	
	2	Manage care in the population board		You are a population leader and must reschedule care for one of the patients.	

^a^A total of 17 scenarios were defined: 11 for patients (eg, login, hotline contact, telecounseling, and forum use), 4 for clinical staff (eg, view agenda and moderate the forum), and 2 for administrative users (eg, population management and create information capsule).

### User Recruitment Phase

A total of 30 individual users were recruited (14 patients, 8 people with a mental health care profile, and 8 people with administrative or logistical activities in the mental health care process). Some of the individuals participated in both evaluation cycles (Table 2).

**Table 2:** Demographic and educational characteristics of participants in the scenario-based usability evaluation of a participatory-designed clinical information system for mood disorder care in Córdoba, Colombia (2022-2023)^a^.

	Average age (years)	Sex			Academic level			
		Male	Female	High school diploma		Technician	Bachelor’s degree	Postgraduate
Patients	24.1	3	11	7		1	4	2
Clinical staff	27.4	1	7	5		0	2	1
Administrative personnel	27.0	1	7	4		0	1	3

^a^A total of 30 participants were included: 14 patients (average age of 24.1 years), 8 clinical staff (27.4 years), and 8 administrative personnel (27.0 years). The table details distribution by sex (male or female) and academic level (high school diploma, technical education, bachelor’s degree, and postgraduate education).

### Implementation Phase and Usability Evaluation by Scenarios

The evaluations were conducted via Microsoft Teams, where users shared their screen. The tests per user lasted approximately 45-90 minutes. Table 3 contains the consolidated number of participants by cycle and their respective roles in the evaluated system.

A total of 6 test groups were conducted (3 types of users in 2 cycles), including the 4 functionalities with the highest scores in the prioritization and impact process: login, providing mental health care and monitoring (telecounseling), assistance to clinical staff in mental health emergencies (hotline), and population management (population dashboard).

**Figure 2:**
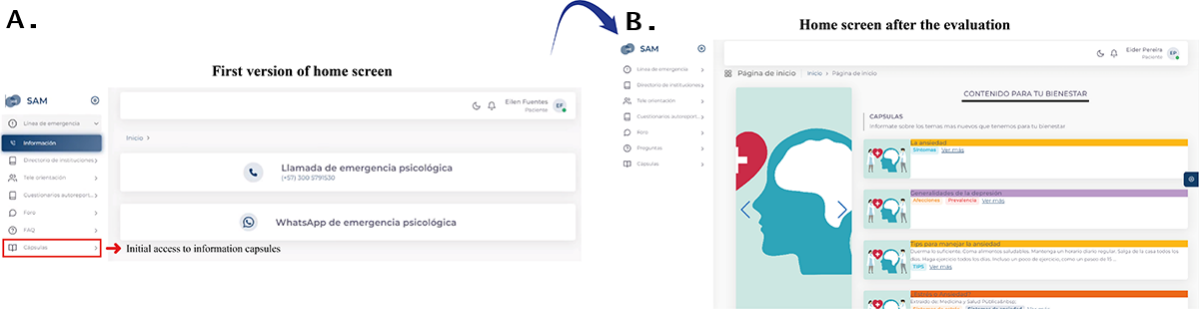
Example of interface changes in the home screen of the clinical information system for mood disorder care, developed through participatory design and evaluated with a scenario-based usability approach in Córdoba, Colombia (2022-2023). The first version (A) offered limited access to information capsules, whereas the revised version (B), following 2 usability testing cycles, presented a redesigned layout highlighting educational resources on symptoms, depression, and mental health promotion.

All events were reported by the researcher (EPM) conducting the test. During the evaluations, 23 critical findings were identified that informed system improvements—9 were system errors and 14 were design errors. System errors refer to malfunctions in the application’s functionality, whereas design errors were limited to issues within the user interface. Figure 2 provides an example of interface modifications based on these findings.

**Table 3:** Distribution of participants by test cycle in the scenario-based usability evaluation of a participatory-designed clinical information system for mood disorder care in Córdoba, Colombia (2022-2023)^a^.

	Cycle 1	Cycle 2	Total (repeated)
Patients	8	7	15 (1)
Clinical staff	4	6	10 (2)
Administrative personnel	4	5	9 (1)
Total participants	16	18	34 (4)

^a^In total, 34 participations were recorded across 2 cycles (16 in cycle 1 and 18 in cycle 2), including patients, clinical staff, and administrative personnel. Four participants took part in both cycles.

#### NASA-TLX Evaluations

A total of 223 individual task evaluations were carried out, where the workload was measured (112 and 111, respectively, in cycles 1 and 2); 165 (74%) were the patient role, 40 (17.9%) clinical staff, and 18 (8.1%) administrative personnel. Multimedia Appendix 1 contains the comparative changes between test cycles 1 and 2 for the scenarios evaluated for all types of users. Multimedia Appendix 1 shows the comparative summary of workload (NASA-TLX) by cycles, functionalities, and types of users. Table 4 shows further details of the components of the workload evaluation by the respective domains of effort.

**Table 4:** Results of workload scenarios (NASA [National Aeronautics and Space Administration] Task Load Index [NASA-TLX]) by domain type in the respective scenarios and test cycles for a participatory-designed clinical system for mood disorder care in Córdoba, Colombia (September-October 2023)^a^.

Type of role and scenario			Cycle		Weighted average mental workload		Weighted Average physical workload		Average temporary workload		Weighted average performance workload		Weighted average effort		Weighted average frustration
Administrative															
	1	1		12.8		0.8		6.8		21.8		10.5		18.0	
	1	2		11.2		0.2		10.8		8.2		6.6		4.2	
	2	1		10.0		0.3		5.3		10.5		9.8		4.0	
	2	2		11.8		0.8		12.6		13.4		12.2		11.0	
Clinical															
	1	1		9.8		5.8		12.5		1.5		4.8		0.8	
	1	2		4.0		2.8		5.5		9.3		3.5		1.3	
	2	1		17.8		4.3		12.5		24.3		15.8		1.0	
	2	2		19.0		3.8		10.3		10.8		13.0		5.3	
	3	1		13.3		0.5		9.3		19.0		6.3		0.5	
	3	2		15.0		9.0		10.3		11.7		13.0		2.7	
	4	1		20.3		0.3		9.8		16.8		12.0		2.0	
	4	2		19.0		3.0		7.5		14.3		8.0		7.7	
Patient															
	1	1		9.3		1.3		9.1		2.4		9.0		8.5	
	1	2		12.7		1.0		8.7		5.7		10.9		5.4	
	2	1		9.9		0.5		16.3		10.8		8.4		15.6	
	2	2		12.0		1.1		9.1		9.0		10.3		11.7	
	3	1		9.8		2.6		15.9		2.4		8.6		4.5	
	3	2		7.1		2.6		9.0		8.0		10.7		8.3	
	4	1		7.6		2.3		19.1		0.9		7.5		1.8	
	4	2		8.3		1.7		6.4		2.4		12.0		11.3	
	5	1		13.5		2.8		21.1		1.9		13.6		8.8	
	5	2		16.3		1.7		7.0		7.4		14.7		10.1	
	6	1		8.8		2.0		15.3		6.0		13.1		8.3	
	6	2		9.0		1.1		9.0		8.1		12.3		12.1	
	7	1		20.1		1.1		11.0		3.8		12.5		5.6	
	7	2		15.4		2.6		12.6		2.1		13.1		7.3	
	8	1		10.1		2.9		14.4		1.4		12.6		2.4	
	8	2		10.9		2.9		7.9		5.9		12.4		4.9	
	9	1		16.3		3.5		14.5		8.3		15.9		3.1	
	9	2		15.6		3.3		6.3		3.7		14.1		8.9	
	10	1		9.5		2.0		18.9		1.5		11.5		0.4	
	10	2		11.3		1.9		8.9		6.1		9.3		1.9	
	11	1		9.5		2.1		19.3		2.1		10.0		0.3	
	11	2		7.6		1.9		8.3		3.6		9.4		2.0	

^a^Detailed breakdown of the workload components, as measured by the NASA-TLX Task Load Index, for the administrative, clinical, and patient user roles across 2 test cycles. Lower values correspond to lower perceived workload and better performance, and higher values correspond to higher perceived workload.

#### PSSUQ Evaluations

Thirty-four usability evaluations were obtained with the PSSUQ tool, which reports a level of usability in global terms by cycles that varied only from 2.2 to 2.3. The results by type of user and their corresponding measurement with the PSSUQ tool showed a tendency in both test cycles toward a better usability rating in the interfaces oriented to clinical and patient users. For example, in cycle 2, there was a difference of 2.0 and 1.9, respectively, versus a score of 3.0 in the administrative interfaces (Tables 5 and 6).

**Table 5:** Overall system usability results, measured by the PSSUQ, for 2 evaluation cycles of a participatory-designed clinical information system for mood disorder care in Córdoba, Colombia (2022-2023)^a^.

	SYSUSE^b^	INFOQUAL^c^	INTERQUAL^d^	Overall
Cycle 1	2.1	2.3	2.2	2.2
Cycle 2	2.2	2.5	2.1	2.3

^a^The Post-Study System Usability Questionnaire scores are presented for each of the 2 test cycles. A low score indicates better usability and greater user satisfaction. The results are broken down into 3 subscales: SYSUSE, INFOQUAL, and INTERQUAL, along with an overall average.

^b^SYSUSE: System Usefulness.

^c^INFOQUAL: Information Quality.

^d^INTERQUAL: Interface Quality.

**Table 6:** Post-Study System Usability Questionnaire, broken down by user role and test cycle of a participatory-designed clinical information system for mood disorder care in Córdoba, Colombia (2022-2023)^a^.

User role	Cycle 1					Cycle 2			
	SYSUSE^b^	INFOQUAL^c^	INTERQUAL^d^	Overall	SYSUSE^b^		INFOQUAL^c^	INTERQUAL^d^	Overall
Administrative	3.5	4.3	3.9	3.9	2.9		3.4	2.7	3.0
Clinical	1.3	1.5	1.3	1.4	2.2		2.1	1.7	2.0
Patient	1.7	1.7	1.9	1.7	1.7		2.2	2.0	1.9

^a^The results compare Post-Study System Usability Questionnaire (PSSUQ) scores across 2 test cycles for administrative, clinical, and patient users. Lower scores on the PSSUQ indicate a better level of usability. The scores are provided for the SYSUSE, INFOQUAL, INTERQUAL, and overall usability.

^b^SYSUSE: System Usefulness.

^c^INFOQUAL: Information Quality.

^d^INTERQUAL: Interface Quality.

## Discussion

### Principal Findings

This study evaluated the usability of a clinical telehealth system for mood disorder care developed through participatory design. The findings show that the system achieved usability scores (PSSUQ 2.2 and 2.3) and improvements in workload between evaluation cycles, particularly for patient-centered tasks, meeting the study’s objective of assessing usability through a scenario-based methodology.

One of the challenges in systems evaluation is the granularity of evaluation at the task level and the order in which such evaluation is performed. The scenario-based evaluation approach allowed planning an evaluation strategy informed by an objective prioritization of functions and tasks. In addition, a separation of user groups was achieved, and common tasks that did not require additional users for validation were identified.

One of the challenges of system evaluation is the granularity of evaluation at the task level and the order in which such evaluation is performed. The scenario-based evaluation approach allowed planning an evaluation strategy informed by an objective prioritization of functions and tasks. In addition, a separation of user groups was achieved, and common tasks that did not require additional users for validation were identified.

The system can be considered to have good usability, given an overall PSSUQ score between 2.2 and 2.3 in cycles 1 and 2, considering that the lower the score, the better the performance. The reference value is 2.8 [20]. These findings suggest that the participatory design process helped anticipate user needs and improve the quality of the product before deployment However, it was also possible to identify elements that previous heuristic evaluations were unable to detect. This confirms how user participation in the design and early usability evaluation contribute to good system usability and help identify opportunities for system improvement before the system goes into production, optimizing the use of development resources. This is consistent with findings from previous studies showing that iterative and participatory usability evaluations with both clinicians and end users can significantly improve system navigation, content structure, and user satisfaction prior to system deployment [22]. This reinforces the importance of iterative usability evaluations early in the system development life cycle to ensure alignment with user needs and task requirements [23].

It is interesting to note how system usability scores were better for users with more functional and task representation. Patients rated the system higher than administrators. This can be explained by the prioritization in the development of interfaces informed by the DO, where development was carried out according to task prioritization. It should also be noted that the differences observed between groups can be attributed to the different types of tasks assigned to each group. Tasks prioritized for patients tended to be simpler and more user-centered, whereas tasks assigned to administrative and clinical users were more complex, involving greater cognitive demands. The differences in scores between cycles 1 and 2 cannot be established as statistically significant given the sample size. However, changes may be due to individual variability. A possible future evaluation method to address this could involve repeated measurements with the same users or increasing the number of evaluations. The number of evaluations may be limited in scenarios where the number of potential users is insufficient. Optional usability evaluations may be implemented in the future for all individuals registered in the mental health program.

When analyzing the sources of task workload, there is an improvement in the NASA-TLX workload index for the vast majority of tasks performed in the second version of the system, except for the forum modeling and population management tasks (associated with clinical users and system administrators). This can be explained by the higher priority given to system changes benefiting patient and family-type users and by the addition of interface elements that had not been subject to group discussion. The most significant source of workload is mental workload, as expected. Future research could focus on improving the information available on-screen to reduce cognitive demands. As anticipated, physical workload is minimal since the interaction does not require extensive clicking.

### Limitations

This study has some limitations. The convenience sample size restricts the generalizability of findings, and the number of evaluations was insufficient to establish statistically significant differences between user groups. In addition, the evaluation of the synchronous interactive telehealth component was not carried out, as this would require a different experimental design. These limitations are consistent with findings from systematic reviews, which report that usability evaluations in mobile mental health interventions often rely on small sample sizes and primarily summative assessment methods [24]. In the future, the mobile interface of the mental health system will be evaluated, as it is considered a highly useful means of accessing and using the mental health system for end users (patients and relatives).

### Conclusions

This study shows that combining participatory design with scenario-based usability evaluation enables early identification of usability challenges and iterative refinement of digital health systems. Beyond the specific case of Córdoba, Colombia, these findings have broader implications: engaging end users early, prioritizing patient-facing functionalities, and conducting iterative usability assessments can enhance adoption, optimize resources, and support sustainable models of mental health care in underserved regions.

## Appendix

Multimedia Appendix 1 Comparative summary of perceived workload (NASA-TLX) across 2 usability test cycles of a participatory-designed clinical information system for mood disorder care in Córdoba, Colombia (2022-2023). The results are shown separately for (A) administrative personnel, (B) clinical staff, and (C) patients, illustrating changes in mental workload associated with different tasks (eg, managing care dashboards, scheduling appointments, forum use, and accessing information capsules). Lower scores represent reduced workload after system adjustments. NASA-TLX: NASA Task Load Index.

## Abbreviations

DO: Domain Ontology
GOMS: Goals, Objectives, Methods, Selection
INFOQUAL: Information Quality
INTERQUAL: Interface Quality
NASA-TLX: National Aeronautics and Space Administration Task Load Index
PSSUQ: Post-Study System Usability Questionnaire
SYSUSE: System Usefulness

## Data Availability

The datasets generated and analyzed during this study are not publicly available due to the sensitive nature of the data, which include recorded usability test sessions with a potential risk of participant identification. To protect participant privacy and confidentiality, access to these data is restricted. Deidentified data derived from questionnaires and usability evaluation forms may be made available from the corresponding author upon reasonable request.
