# Supplementary figures and images for: Clinical System for Mood Disorder Care in Córdoba, Colombia: Participatory Design and Scenario-Based Usability Evaluation Study
Source: JMIR Form Res. 2025 Oct 6;9:e58909. doi: 10.2196/58909 (PMC12538187; doi:10.2196/58909)

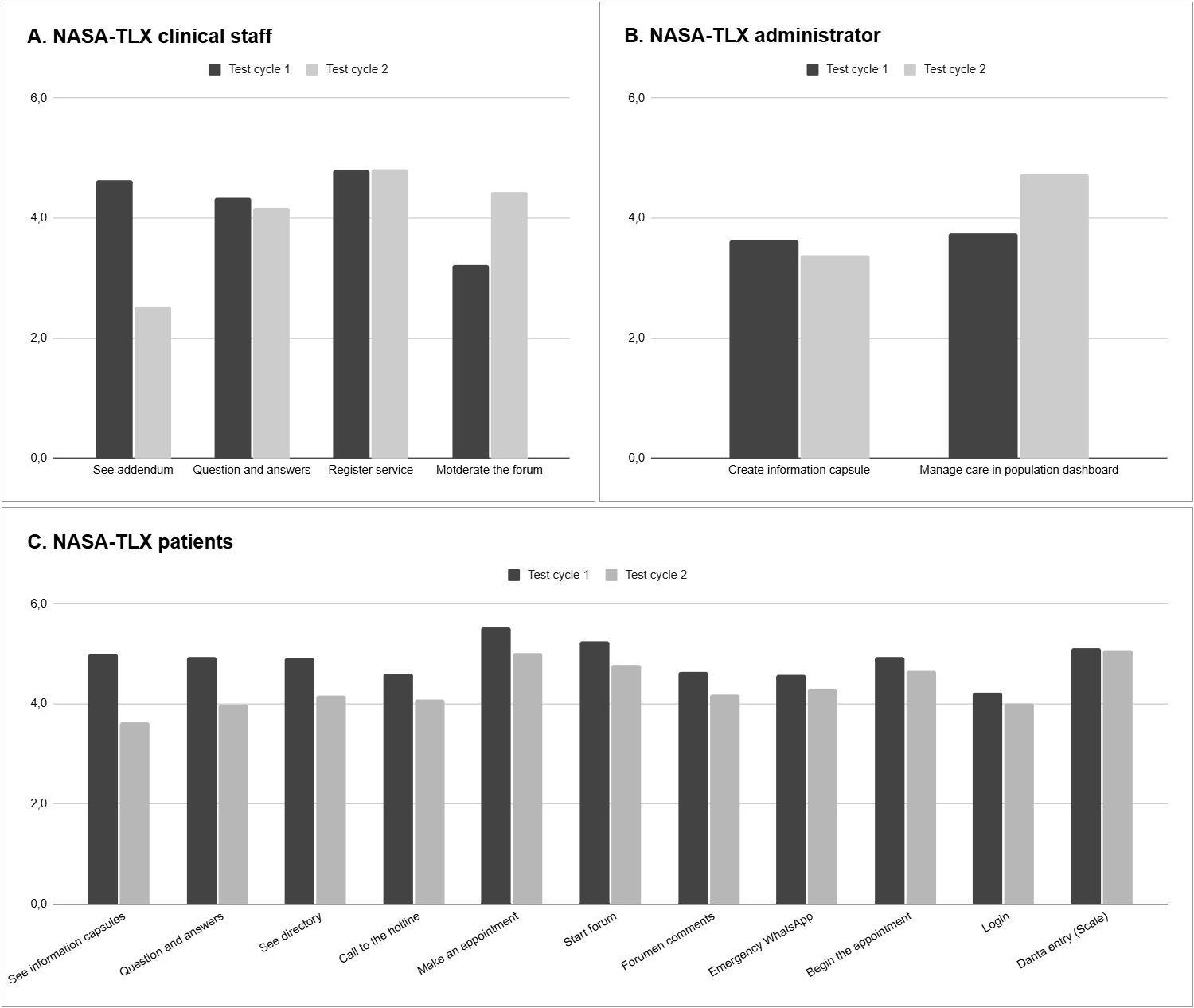

Supplement: Multimedia Appendix 1 [file formative_v9i1e58909_app1.png]
